# Overview of Phage Defense Systems in Bacteria and Their Applications

**DOI:** 10.3390/ijms252413316

**Published:** 2024-12-12

**Authors:** Xiaomei Xu, Pengfei Gu

**Affiliations:** School of Biological Science and Technology, University of Jinan, Jinan 250022, China; 15269800127@163.com

**Keywords:** phage, bacteria, anti-phage mechanism

## Abstract

As natural parasites of bacteria, phages have greatly contributed to bacterial evolution owing to their persistent threat. Diverse phage resistance systems have been developed in bacteria during the coevolutionary process with phages. Conversely, phage contamination has a devastating effect on microbial fermentation, resulting in fermentation failure and substantial economic loss. Accordingly, natural defense systems derived from bacteria can be employed to obtain robust phage-resistant host cells that can overcome the threats posed by bacteriophages during industrial bacterial processes. In this review, diverse phage resistance mechanisms, including the remarkable research progress and potential applications, are systematically summarized. In addition, the development prospects and challenges of phage-resistant bacteria are discussed. This review provides a useful reference for developing phage-resistant bacteria.

## 1. Introduction

Bacteriophages represent the most predominant organism on earth; they can hijack the biosynthetic machinery of host cells for their multiplication and progeny release. It was estimated the quantity of phages is ten times more than that of bacteria [1]. Phages are currently classified into 314 families based on their genomic features by the International Committee on Taxonomy of Viruses (ICTV) [2]. Alternatively, phages can also be classified into virulent and temperate phages according to their lifestyle. Virulent phages can lyse bacteria, and their life cycles include adsorption, DNA or RNA injection, propagation, and release. Conversely, temperate phages do not cause bacterial cell lysis; however, they integrate their own DNA into the genome of the host bacterium and pass it on to the next generation with the proliferation of the host bacterium [3].

Phages, especially virulent phages, pose a notable threat to bacteria because of their high specificity and infestation efficiency. Bacteria and phages have coevolved in a perpetual battle, resulting in sophisticated mechanisms in phages for manipulating their hosts and an equally diverse set of phage defense systems in bacteria for withstanding phage predation [4]. Under persistent survival pressure, bacteria not only evolved versatile phage-resistant strategies at the single-cell level, but also developed complex collaborative mechanisms in potential multicellular community behavior. These defense mechanisms can precisely recognize and defend against bacteriophages independently or synergistically [5,6].

At the single-cell level, the bacterial defense system can deploy refined defenses based on the bacteriophage lifecycle, such as blocking phage adsorption, preventing the injection of phage genetic material, and interfering with DNA replication, protein shell synthesis, and assembly in progeny bacteriophages. Once phages break through all defense systems, bacteria often trigger the suicide mechanism before the release of phage progeny, thus reducing the potential threat to the surrounding neighboring cells [7]. Considering that phage contamination has become a persistent problem in bacteria-based industrial biotechnology processes, different phage-resistant strains have been developed according to natural anti-phage mechanisms [8]. In addition, genetic elements derived from phage-resistant systems, such as the clustered regularly interspaced short palindromic repeat-associated protein (CRISPR-Cas) system, have been employed to develop effective gene-editing tools [9]. Phages have been also considered as potential candidates for therapeutic treatments of bacterial infections (commonly referred to as “phage therapy”), which was pioneered by Felix Herelle nearly a century ago and is still being explored and emphasized by the medical community [10]. In addition, phage therapeutics have become an effective solution for infection resulting from drug-resistant bacteria [11].

In this review, the anti-phage systems of bacteria throughout the phage life cycle at the unicellular and multicellular levels are introduced in detail. Moreover, the prospects and challenges associated with the development of phage-resistant bacterial strains have been addressed. We expect that this review will provide a reference for designing more robust and broad anti-phage host cells by integrating multiple defense mechanisms, which can partly address the challenges of bacterial phage contamination in the industrial fermentation of high-value-added chemicals.

## 2. Anti-Phage Mechanisms at the Single-Cell Level

### 2.1. Adsorption Inhibition

**Figure 1:**
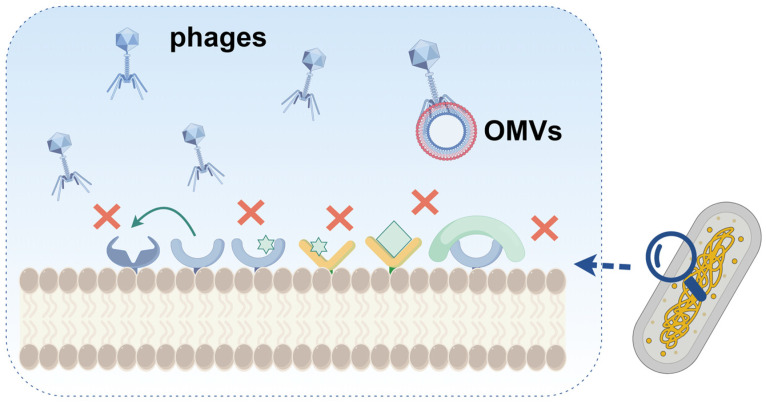
Adsorption inhibition mechanism of bacteria against phages.

The first step in phage infestation is adsorption onto the microbial cell membranes. Barrier protection of the cell membrane is the first line of defense against phages (Figure 1). Fluorescent labeling of cells and phages indicates that the biofilm structure serves as a barrier, preventing phages from entering the interior of the biofilm [6]. Gram-negative bacteria secrete outer membrane vesicles (OMVs) that inhibit phage binding to the host cell. For example, Reyes-Robles et al. observed that *Vibrio cholerae* secretes outer membrane vesicles that can bind to three different phages, ICP1, ICP2, and ICP3, depending on the phage receptor type on the OMVs [12]. To initiate the infection process, phage tails recognize specific cell surface receptors, such as capsule polysaccharides (CPSs) and lipopolysaccharides (LPSs) [13,14]. Alternatively, bacteria can thwart adsorption by modifying surface receptors. Hanjeong et al. showed that the type IV pili of *Pseudomonas aeruginosa* can be modified by glycosylation, which can protect it against certain filament-specific phages [15]. In *Listeria monocytogenes*, loss or mutation of wall teichoic acid rhamnosylation increases the resistance to multiple phages [16]. Scholl et al. showed that shielding the phage receptor with surface structures (e.g., capsular polysaccharide pods) similarly blocks phage adsorption [17]. In addition, mutations or nonexpression of phage receptor genes can prevent phage binding. Burmeister et al. mutated *tolC* and lipopolysaccharide (LPS) genes in *Escherichia coli* and enhanced the resistance of the strain to the bacteriophage U136B. However, the lack of TolC, a key component of the antibiotic efflux pump, renders *E. coli* susceptible to a wide range of antibiotics [18]. Similarly, Xuan et al. revealed that a key component of type IV pili(T4P), a common phage receptor, is mutated in the multidrug-resistant (MDR) strain *P*. *aeruginosa* variant PAO1-R1. This mutation effectively prevented the adsorption of the phages vB_Pae_QDWS and vB_Pae_W3, thereby conferring phage resistance. However, this modification re-sensitized the MDR strain to gentamicin and polymyxin B [19], providing important clinical implications for the treatment of pathogenic drug-resistant bacteria.

In nature, three phages, T1, T5, and φ80, employ the same ferricrome transporter FhuA on the outer membrane of *E. coli* as a receptor for DNA injection [20]. Accordingly, the inactivation of FhuA improves the resistance of *E. coli* to these three phages [8]. Alternatively, some bacteria can synthesize proteins that competitively bind to phage receptors, thereby inhibiting phage adsorption. The outer membrane protein A (OmpA) is an adsorption receptor for various *E. coli* phages [21] and the outer membrane lipoprotein encoded by *traT* is located on the F plasmid. When the F plasmid is expressed in *E. coli*, TraT will interact with OmpA to inhibit phage attachment [22]. Sorek et al. identified a novel phage-resistant defense system in bacteria that consists of the ubiquitin-like proteins E1 and E2. During phage infection, this system can specifically bind ubiquitin-like proteins to the phage central tail fibers, blocking the central tail fibers and preventing daughter phages from recognizing the host receptor [23].

### 2.2. Blocking DNA Injection

**Figure 2:**
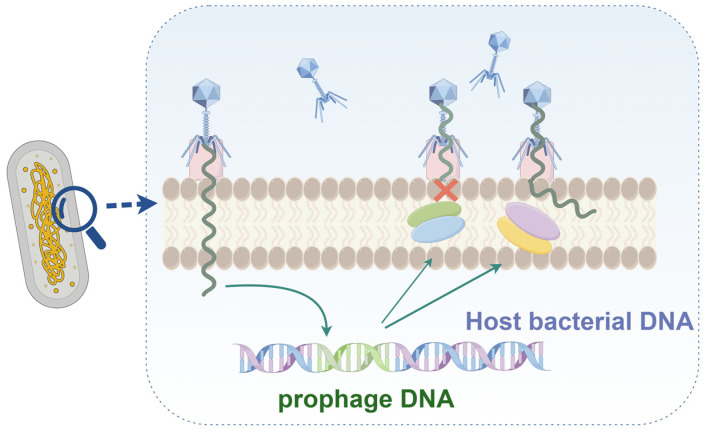
The Superinfection Exclusion (SIE) system, which can interfere with the injection of phage DNA.

Even if the phage is successfully adsorbed onto the host cell, the Superinfection Exclusion (SIE) system can interfere with the injection of its DNA (Figure 2). SIE can function in the early stages of phage infection by preventing further phage adsorption or in the later stages of infection by preventing the injection of phage DNA [24]. As proteins involved in SIE are usually located in prophages, SIE is usually classified as a phage–phage interaction system. SIE proteins are mostly membrane-anchored proteins or are associated with membrane components [25]. Filamentous prophages in *P*. *aeruginosa* PAO1 are super-infective phage virion particles. Wang et al. found that structural proteins encoded by filamentous prophages can exclude superinfected Pf phages by interfering with type IV pili (T4P). Specifically, the phase minor capsid protein pVII of the phage inhibits Pf phage adsorption by interacting with PilC and PilJ of T4P. In addition, pVII can protect host cells from pili-dependent lysosome infection, whereas pIlI can protect host cells from pili-independent lysosome infection [26]. After infection with T4 bacteriophages, bacterial cells rapidly exhibit resistance to the same or similar types of bacteriophages. Shi et al. found that the periplasmic protein Spackle can form a stable complex with the lysozyme domain of the T4 phage tail spike protein, gp5. Fluorescence-based assays indicated that Spackle effectively inhibited lysozyme activity and prevented the translocation of lysozyme DNA into the cytoplasm of host cells, thereby protecting against virulent phages. The crystal structure of the Spackle–gp5 lysozyme complex indicates that the inhibitory effect of Spackle is facilitated by a conformational shift in the active site of lysozyme and partial obstruction of its oligosaccharide-binding cleft [27]. In addition, the *ltp* gene is derived from the temperate bacteriophage TP-J34 of *Streptococcus thermophilus*. Sun et al. expressed *ltp* in *S. thermophilus* J34-6f, resulting in successful interference with the bacteriophage TP-J34. Similarly, the expression of *ltp* in *Lactococcus lactis* Bu2-60 demonstrated that the same repeated infection exclusion system worked well against bacteriophage P008 [28]. SIE can also affect viral evolution. Hunter et al. used stochastic simulations to demonstrate that repeated infection exclusion systems negatively affect the long-term adaptation of viral populations [29].

### 2.3. Restriction–Modification Systems

When bacterial cells are unable to prevent the injection of phage DNA, multiple internal defense systems will start. Most phage defense systems consist of two essential elements: a sensor for detecting infection and an effector that either targets the phage or eliminates the infected host prior to phage reproduction cycle completion, thereby effectively safeguarding the remaining cellular population from newly released viral particles [30]. As multiple antiviral systems can coexist in a single strain, a new tool for the automated detection of antiviral systems in prokaryotic genomes, Defense Finder, was developed by Tesson et al. [31]. They used this tool to examine all known antiviral systems in a database containing more than 21,000 complete microbial genomes, and their analysis showed that the pool of antiviral systems in prokaryotes is highly variable and species-specific. They also showed that the restriction–modification (RM) system was widespread and present in 84% of bacterial genomes, followed by the CRISPR-Cas system in 40% of the bacterial genomes.

**Figure 3:**
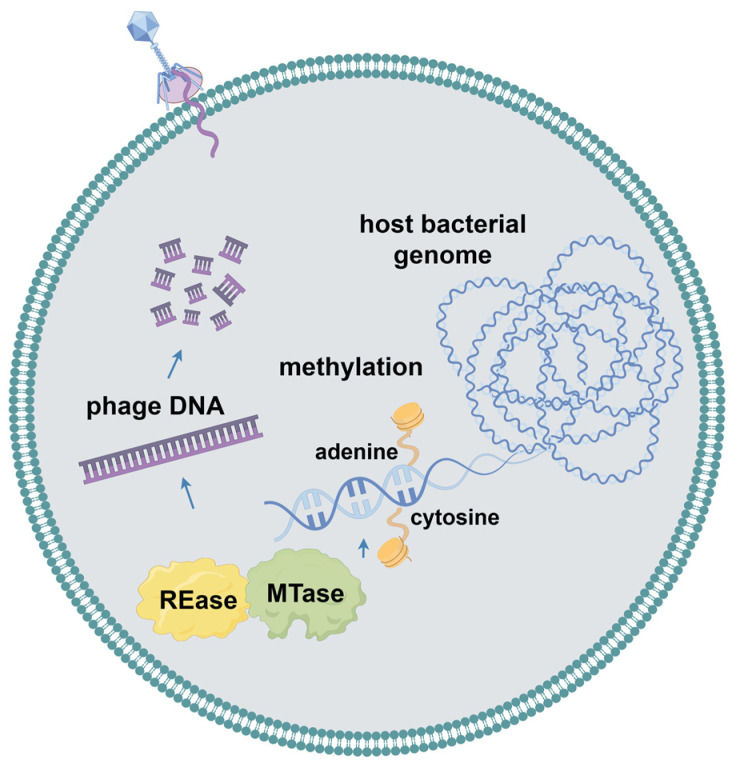
The mechanism of the restriction–modification (RM) system in bacteria.

The RM system serves as an innate immune mechanism in bacteria and protects against foreign DNA (Figure 3). Two enzymes with different activities, methyltransferases (MTases) and restriction endonucleases (REases), play major roles in the RM system [6]. MTases transfer a methyl group to both strands and methylate the adenine or cytosine residues of their own DNA. Subsequently, REases recognize and cleave identical unmethylated DNA sequences [32]. As a result, host cells protect their genomes from recognition by modifying their DNA through methyltransferases. The invading phage, which is typically unmethylated, is cleaved upon injection into the host. Traditionally, RM systems have been categorized into four types based on subunit composition, recognition sequences, DNA cleavage positions, cofactor requirements, and substrate specificity. Type I and III RM systems cleave and methylate translocated DNA far from the recognition site. Type II is the most prevalent RM system, and it cleaves DNA at or near the recognition sites. Unlike other RM systems, type IV systems contain restriction endonucleases or methylases that cleave modified or unmodified DNA [33]. However, some phages can evade recognition by RM systems by utilizing alternative modified bases such as uracil, hydroxymethyluracil, and hydroxymethylcytosine (hmC). For example, the T4 phage incorporates 5-hmC into its DNA to escape host killing. Additionally, type IV RM systems can protect bacteria from phages escaping from other RM systems. Interestingly, the T4 phage then gains resistance to the type IV RM system again through the glycosylation of HMC residues, representing a coevolution process of bacteria and phages [34].

Apart from the RM system itself, other defense systems with functions similar to RM also exist in bacteria, which can recognize and defend against the invasion of foreign nucleic acids by methylating host DNA. Goldfarb et al. reported the presence of a six-gene cassette in the genome of *Bacillus cereus*, which showed resistance to both virulent and mild phages, including a putative Lon-like protease, an alkaline phosphatase structural domain protein, a putative RNA binding protein, a DNA methylase, an ATPase structural domain protein, and an A protein of unknown function. This new defense system, called Bacteriophage Exclusion (BREX), allows the phage to adsorb and inject DNA but prevents phage DNA replication [35]. BREX can undergo methylation modifications at the TAGGAG motifs in bacterial genomes to direct self- and non-self-recognition. However, unlike RM systems, phage DNA does not appear to be cleaved or degraded by BREX, suggesting that BREX may inhibit phage proliferation and represent a novel defense mechanism. Gordeeva et al. investigated the role of the BREX system in wild *E. coli* and they found that BREX enables cells to be resistant to λ phage infection; however, the induction of λ prophage from BREX-carrying cells also leads to the production of viruses that overcome this defense [36]. Ofir et al. reported a similar defense system named the defense island system associated with restriction–modification (DISARM), which consists of five genes, including a DNA methylase and four other genes annotated as helicase structural, phospholipase, DUF1998 structural domains and a gene of unknown function [37]. DISARM can modify CCWGG motifs in the host genome, and its heterologous expression greatly improves phage resistance in *Bacillus subtilis*. Similar to the BREX system, the DISARM system also prevents phage DNA replication. Both BREX and DISARM systems target only phage DNA and have no restrictions on the injection of exogenous plasmid DNA into host cells [35,37]. However, the precise defense mechanisms of these two systems remain largely unelucidated.

In nature, epigenetic modifications occur in the DNA of all organisms by the addition of various chemical groups to the bases of nucleic acids, ranging from methyl groups to polyamines, amino acids, and sugars [38]. These modified bases can provide nucleic acids with additional functions such as protection and genetic regulation. Recently, Wang et al. observed that the pentose phosphate backbone of nucleic acids can also be modified by phosphorothionylation, including the substitution of sulfur for a non-bridging oxygen on the pentose phosphate backbone [39]. This oxygen–sulfur exchange is catalyzed by DndABCDE, which often constitutes a defense barrier in bacteria with DndFGH and acts as a restriction effector to discriminate and attack non-PT-modified exogenous DNA. Xiong et al. found a similar defense system based on phosphorothioate modification in archaea, in which the DndCDEA cluster specifically modifies DNA with phosphorothioate, and PbeABCD acts as a restriction element to prevent viral transmission by inhibiting DNA replication. In contrast to DndFGH in bacteria, DndCDEA-PbeABCD does not degrade or cleave viral DNA [40]. In addition to the DndACDE system, Xiong et al. have identified an unusual phosphorothionylation modification system, SspABCDE, in *Streptomyces yokosukanensis, Vibrio cyclitrophicus,* and *E*. *coli*. SspABCD can modify a single strand of host DNA via high-frequency phosphorothionylation. Among these proteins, SspB possibly acts as a nickelase by introducing nickel to facilitate sulfur binding [41]. Stimulated by NTPase activity, SspE recognizes specific modifying sequences and introduces nicks and damages phage DNA, thereby inhibiting DNA replication and ultimately preventing phage propagation. Wang et al. found that SspABCD, in addition to being coupled to SspE for restriction–modification, could also be coupled to SspFGH for anti-phage activity [42]. SspABCD-SspFGH phosphorylates the DNA backbone and destroys the unmodified DNA. The defense mechanisms of SspFGH and SspE are slightly different. SspE has anti-phage activity only in the presence of DNA containing the 5′-C_PS_CA-3′ modification; however, it exhibited no activity in PT-deficient mutants. Conversely, SspFGH can indiscriminately target non-Pt-modified DNA. However, SspFGH and SspE are compatible, and the modified 5′-CPSCA-3′ can bind to both SspFGH and SspE, which greatly enhances resistance to phages. Although PT-based and methylation-based restriction–modification systems demonstrate similar defense mechanisms against exogenous DNA, they exhibit different target preferences against invaders, which can employ distinct recognition sequences within a host strain and facilitate host cell defense against more types of phages [43].

### 2.4. CRISPR-Cas Systems

**Figure 4:**
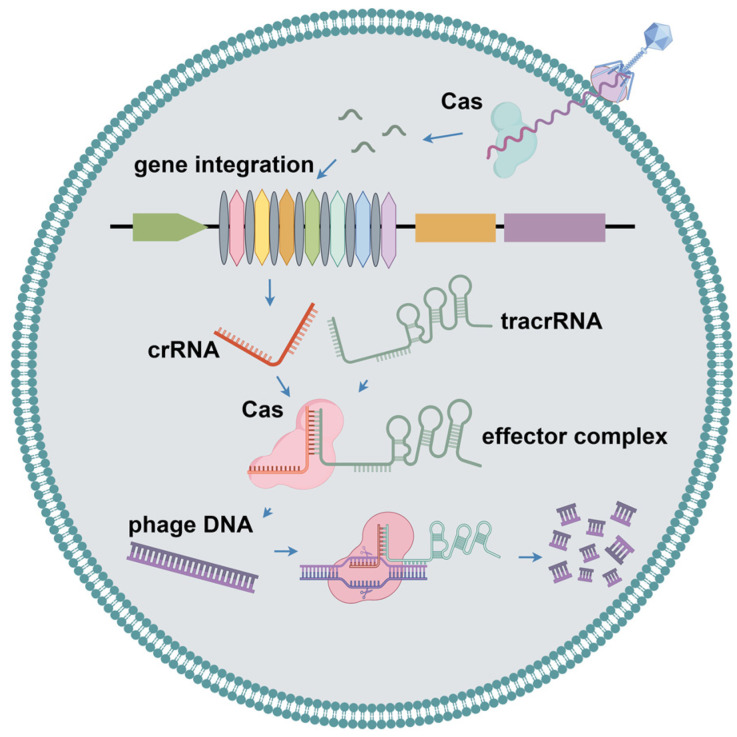
The mechanism of the CRISPR-Cas system in bacteria.

CRISPR and CRISPR-associated Cas proteins in bacteria and archaea constitute the CRISPR-Cas system (Figure 4). This system drives immune responses, including adaptation, CRISPR RNA (crRNA) biosynthesis, and interference. During the adaptation process, foreign invasive nucleic acids are selected, processed, and incorporated into CRISPR spacer sequences, thereby establishing a memory of past infections. When exogenous DNA reappears, crRNA is generated by the transcription of the spacer sequence and coordinates with the Cas protein to recognize exogenous DNA and cleave it by complementary base pairing [44]. The CRISPR system is categorized into two types and six subtypes, based primarily on the composition of Cas genes [45]. Class 1 CRISPR-Cas systems (types I, III, and IV) use multiple subunit effector complexes for interference, whereas class 2 systems (types II, V, and VI) use single-protein effector modules for interference [46]. Nami et al. analyzed the genome sequence of *Lactobacillus yoelii* and found significant differences between CRISPR-Cas systems derived from different strains [47]. Deng et al. conducted a systematic analysis of CRISPR-Cas systems and prophages in *Candidatus* Accumulibacter, indicating the CRISPR-Cas systems of different Ca. Accumulibacter members were not inherited from their last common ancestor, but rather acquired from different lineage members at different nodes through horizontal gene transfer [48].

In addition, the CRISPR-Cas system, which is prevalent in bacteria and archaea, can be precisely regulated; however, it does not induce self-cutting. Lin et al. identified an intrinsic negative modulator, CdpR, which can inhibit intracellular signaling to block the transcription of Cas proteins [49]. CdpR-mediated LasI/RhlI/Vfr intracellular signaling inhibits the cleavage of endogenous bacterial sequences by blocking the RNA cleavage activity of Cas3. Thus, CdpR has an important inhibitory effect on the CRISPR-Cas system to avoid possible self-cutting; however, it may increase the risk of infection with exogenous nucleic acids. Recently, Maguin et al. identified a novel anti-phage defense system including cooperating RM and CRISPR-Cas [50]. In this system, the RM system cuts viral DNA to produce double-strand breaks (DSBs), which help the CRISPR-Cas system obtain new spacer sequences from inactivated phage DNA and enable long-term specific immunity.

### 2.5. Abortive Infection

**Figure 5:**
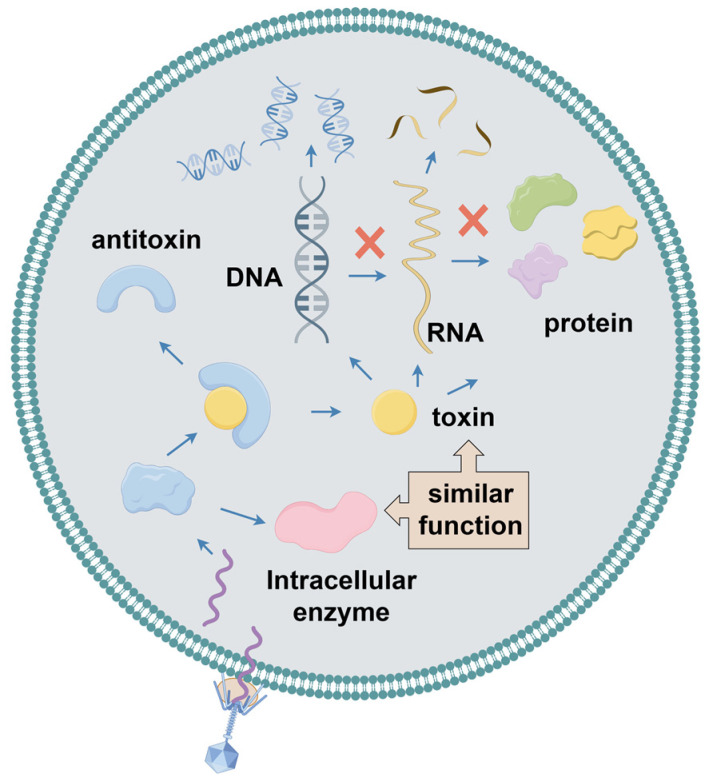
Abortive infection (Abi) system and toxin–anti-toxin (TA) system in bacteria.

Abortive infection (Abi) is another common phage defense strategy that represents cellular self-sacrifice (Figure 5). In detail, infected cells protect bacterial colonies by committing suicide before the phage completes its life cycle, preventing the release of synthesized progeny phages and the spread of phages [51]. The Abi system is capable of sensing phage genome replication, early or late phage structural proteins, phage proteins expressed in the cytosol during replication, a wide range of phage DNA transcripts, and the phage-mediated shutdown of host gene expression [33]. Depardieu et al. identified a serine/threonine kinase, Stk2, in *Staphylococcus aureus*. After phage DNA is injected into host cells, Stk2 kinase is activated, leading to the phosphorylation of several proteins involved in translation, global transcription control, cell cycle control, stress response, DNA topology, DNA repair, and central metabolism within the host cell, finally resulting in cell death due to extensive phosphorylation within the cell [52]. Prokaryotic argonautes (pAgos) are also involved in phage defense by recognizing invading exogenous nucleic acids. Most pAgos are short (including only the MID and PIWI domains) and typically interact with proteins containing Sir2, Mrr, or TIR domains. Defense-associated sirtuins (DSRs) comprise a family of proteins that typically possess a sirtuin (SIR2) structural domain at the N-terminus [53]. Zaremba et al. have shown that a short pAgo forms a stable heterodimeric complex with Sir2 proteins bound to NAD in *Geobacter sulfurreducens* [54]. The GsSir2/Ago complex may recognize the invading exogenous DNA and activate the Sir2 subunit. The activated GsSir2/Ago complex can then act as an NADase to hydrolyze NAD into ADPR and trigger endogenous NAD depletion, resulting in cell death and preventing the proliferation of invading DNA. This interaction triggers an immune-sensing mechanism that activates Abi, thereby providing robust antiviral protection to the cells. Sather et al. identified a single-phage defense protein, Hna, in *Sinorhizobium meliloti*. When exogenous nucleic acids invade the cell, Hna is activated by phage DNA-binding proteins and triggers an anti-host response that results in the death of infected cells without the release of phage progeny [55]. The Abi system can also be combined with the CRISPR-Cas system to exert a strong anti-phage effect. Mayo-Muñoz et al. demonstrated that the type III CRISPR-Cas system recognizes phage mRNA exported from the nucleus, which activates NucC, a cyclic triadenylate-dependent accessory nuclease. Although the CRISPR-Cas system cannot access the phage DNA in the nucleus, it degrades bacterial chromosomes, inhibits phage maturation and replication, and triggers cell death. Thus, type III CRISPR-Cas-mediated immunization against phages occurs via Abi [56]. In general, the death of host cells during phage infection is probably not due to the Abi activation mechanism, but rather due to extensive and irreversible damage to host genome by phages. However, Abi defense strategies will start once the phage infection is extremely difficult to control or phages exhibit resistance to other host defenses [30].

Retrons in bacteria typically consist of a reverse transcriptase (RT) and non-coding RNA (ncRNA). Using ncRNA as a template, RT can synthesize chimeric RNA/DNA molecules in which the RNA and DNA components are covalently bonded together. Millman et al. reported that retrons can act as an anti-phage defense system, including three components, RT, ncRNA, and effector proteins, which confer cellular defense against a broad spectrum of phages through Abi [57]. In *E. coli*, the reverse transcript Ec48 can monitor the activity of the RecBCD complex, implying that inhibition of RecBCD triggers systemic activity [57]. Bobonis et al. showed that Retron-Sen2 in *Salmonella typhimurium* encodes an accessory toxin protein, RcaT, that is neutralized by the reverse transcriptase–msDNA anti-toxin complex. Accordingly, the RcaT-containing retron family constitutes a novel tripartite DNA toxin–anti-toxin system. Phage-associated triggers can act directly on msDNA to disrupt msDNA biosynthesis, thereby activating RcaT which resists phage infection by aborting the infection [58].

In contrast, DNA and retroviruses consume large amounts of deoxyribonucleotides during replication. Tal et al. identified a family of bacterial defense deaminases that degrade dCTP and dGTP. During phage infection, bacterial defense proteins deplete the nucleotide pool of specific deoxyribonucleotides (dCTP or dGTP), thereby depriving the supply of essential phage DNA and halting its replication [59].

The cyclic GMP-AMP synthase (cGAS)–STING pathway is a pivotal component of the autonomous innate immune system in animal cells [60]. The cGAS protein functions as a sensor for cytoplasmic viral DNA. Upon detecting this DNA, it synthesizes a cyclic GMP-AMP (cGAMP) signaling molecule, which then binds to the STING protein, triggering immune response activation [61]. Davies et al. found that cGAMP is also present in bacteria [62]. Phage infection of bacterial cells directs the cell to synthesize cGAMP and activates phospholipases, which cause the loss of cell membrane integrity and lead to cell death [61]. This is also a mechanism of Abi, as cell death occurs before the completion of phage reproduction [63,64]. The subtlety of this defense mechanism, creatively named CBASS (cyclic oligonucleotide-based anti-phage signaling system), contains at least two core protein components. The first protein acts as a keen sentinel, precisely detecting phage invasion, and subsequently triggering the generation of a cyclic oligonucleotide signal that serves as an alarm system. The other functions as an effector executor that combines the transmembrane function, nucleic acid endonuclease activity, and TIR structural domains, which can acutely capture these cyclic oligonucleotide signals and decisively activate the cell-killing mechanism. This sophisticated cascade of responses ensures that the metabolic activities of the infected bacteria are rapidly and effectively blocked before the phage completes its replication cycle, thereby effectively fending off the phage [65,66]. All CBASS manipulators incorporate a unique cGAS/DncV-like nucleotidyl transferase (CD NTase) that detects subtle signs of phage replication. Upon sensing a threat, the CBASS rapidly activates its catalytic function to synthesize key nucleotide secondary messenger signals. Cyclic dinucleotides (CDNs) often use purine and pyrimidine nucleotides as raw materials to initiate antiviral defense [64]. Millman et al. successfully identified and delineated four major CBASS types through an in-depth exploration and fine-grained resolution of the manipulator organization [66]. The type I CBASS comprises only two parts: an oligonucleotide cyclase and effector genes. Conversely, the type II CBASS covers auxiliary genes encoding ubiquitin-associated structural domains in addition to core cyclase effector proteins, and these genes are referred to as *Cap2* and *Cap3*. The type III CBASS includes auxiliary genes encoding the structural domains of HORMA and TRIP13, which are referred to as *Cap7* and *Cap6*, respectively. The TRIP13 protein has the function of inhibiting the activity of the HORMA protein [67]. The type IV CBASS contains auxiliary proteins involved in nucleotide modifications [66]. Lowey et al. identified a class of CBASS transmembrane (TM) effector proteins, the Yersini TM effector Cap15, which possesses a cyclic dinucleotide receptor domain activated by antiviral nucleotide signals and undergoes oligomerization. Activated Cap15 re-localizes throughout the cell and induces endosomal rupture. This process limits phage proliferation by inducing cell death [68]. The CBASS can also synergize with the CRISPR-Cas system. Lau et al. showed that the CBASS effector, NucC, associates with restriction endonucleases and uniquely assembles to form a homotrimeric structure. The NucC trimer promotes the assembly of NucC homohexamers upon binding to a cyclic triadenylate second messenger. This hexamer can cleave nonspecific double-stranded DNA. In infected cells, the activation of NucC leads to complete disruption of the bacterial genome, which in turn triggers cell death before the completion of phage replication. In addition to the CBASS, Lau et al. identified NucC homologs in several type III CRISPR/Cas systems that may act as auxiliary nucleases activated by cyclic oligoadenylate secondary messengers synthesized by the effector complexes of these systems [69]. Ye et al. have identified a similar phage immune pathway in various bacteria. This pathway employs HORMA structural domain proteins to identify specific peptides that bind to and activate cGAS/DncV-like nucleotidyltransferase (CD-NTase), triggering the production of cyclic adenosine triphosphate adenylate (cAAA) as a secondary messenger. The nucleic endonuclease effector NucC is then stimulated, resulting in bacterial DNA damage and ultimately cell death [67]. Currently, research on the CBASS has mainly focused on gaining a deeper understanding of its mechanism by analyzing the structures of related proteins.

In addition, Gao et al. identified a new phage defense system, RADA, which consists of two components, adenosine triphosphatase (RdrA) and adenosine deaminase (RdrB), and is capable of effectively defending against a wide range of dsDNA phages, including T2, T3, T4, and T5 [70]. However, some variants also contain a small membrane protein, the SLATT structural domain, or the type VI-B CRISPR accessory protein Csx27 [71,72]. The defense activity of the RADAR system occurs primarily during the early stages of the phage infection cycle. Once phages occur, adenosine deaminase catalyzes adenine deamidation [70]. Currently, research on the RADAR system mainly focuses on analyzing the macromolecular structures formed by the assembly of RADAR-associated proteins and attempts to explain how these assemblies can effectively hinder the phage process at the structural level [73,74].

### 2.6. Toxin–Anti-Toxin System

In archaea and bacteria, the toxin–anti-toxin (TA) system encodes a toxin that interferes with cellular processes, thereby inhibiting cell growth (Figure 5). It also encodes an anti-toxin that protects the cell from the toxin [75]. Toxins are highly stable and inhibit several key cellular physiological activities, including cell division, transcription, translation, replication, and maintenance of membrane integrity. However, anti-toxins are less stable. The TA system plays crucial roles in maintaining plasmid stability, inhibiting phages, facilitating biofilm formation, responding to stress, and regulating cell death. However, its primary role is to provide anti-phage defense [76]. In addition, the CRISPR/Cas system components can be acquired using TA systems [77]. Currently, TA systems are categorized into eight types according to the detailed mechanism of the anti-toxin involved [78]. In type I TA systems, non-coding small RNA anti-toxins act as antisense RNAs that bind to toxin-encoded mRNAs and inhibit their translation. Conversely, in type II TA systems, the anti-toxin directly interacts with its cognate toxin via protein–protein binding, thereby neutralizing it and forming a TA complex. In type III TA systems, the anti-toxin is an RNA molecule that directly binds to toxin proteins and effectively neutralizes their toxicity. In the type IV TA system, the anti-toxin counteracts the activity of the toxin by interacting with its target. In type V TA systems, the anti-toxin GhoS functions as a specific RNase that degrades toxin mRNA. In contrast, in type VI TA systems, the anti-toxin protein serves as a proteolytic adapter, stimulating the degradation of the toxin SocA. In type VII TA systems, the anti-toxin neutralizes toxin proteins through chemical modifications. Finally, in type VIII TA systems, the small RNA toxin CreT sequesters tRNAUCU, whereas the crRNA-like anti-toxin CreA directs the transcription of Cas proteins to inhibit the CreT toxin [76]. Guegler et al. characterized a type III TA system, ToxIN, which is induced upon infection of *E. coli* with various phages and leads to the loss of the intrinsically unstable ToxI anti-toxin. Subsequently, the intracellular ribonucleic acid endonuclease ToxN is activated, effectively hindering phage development by cleaving the viral mRNA and suppressing its translation [79]. Sometimes, when a host cell is infected by a phage, it activates specific TA systems that provide the cell with resistance to the phage by aborting the infection mechanism [80]. Cui et al. observed that *phi3T*_93, located in a functionally conserved operon, was expressed and bound to the anti-toxin MazE in the MazF/MazE toxin–anti-toxin (TA) module when the SP beta-like phage infected host cells, thereby promoting MazF toxicity. This process inhibited phage lysis and proliferation via Abi [81]. Songailiene et al. demonstrated an HEPN-MNT TA system from cyanobacterium *Aphanizomenon* located adjacent to the I-D CRISPR-Cas system. The HEPN in this system is a toxin RNase that has the ability to cleave four nucleotides (nt) at the 3′ end of tRNA, thereby interfering with the translation process. The MNT anti-toxin can inactivate the HEPN toxin through covalent diAMPylation. Based on these observations, Songailiene et al. proposed that the HEPN-MNT system acts as an intracellular ATP sensor capable of monitoring ATP homeostasis and releasing active HEPN toxins when ATP levels are low [82]. In addition, Hoskisson et al. characterized an intricate and novel phage restriction system in *Streptomyces coelicolor* named Pgl (phage growth restriction system). This system involves a toxin/anti-toxin mechanism, in which the toxic protein PglX, with DNA methyltransferase activity, is activated in the absence of the functional anti-toxin PglZ. Moreover, the ATPase activity of PglY and protein kinase activity of PglW are indispensable for conferring phage resistance in the Pgl system. When phage φC31 infects cells containing the Pgl system, PglW may exhibit phage resistance by phosphorylating signals to other Pgl proteins, which in turn activate the DNA methyltransferase PglX [83].

The TA system not only provides host cells with resistance to exogenous nucleic acids but also maintains the stability of heterologous nucleic acids. Czarnecki et al. found that plasmid pKON1 in *Paracoccus kondratievae* NCIMB 13773T carried the *hipAB* family toxin/anti-toxin system, which is important for the stable maintenance of pKON1 [84]. Chen et al. utilized a newly developed TA system to successfully develop an antibiotic-free expression plasmid vector, which may have applications in nonviral gene therapy and DNA vaccine development [85]. Bleriot et al. investigated the specific role of the type II TA system PemIK (PemK/PemI) in phage inhibition and revealed that the overexpression of the PemK toxin induces bacteria to enter a dormant state, thereby effectively inhibiting phage infections [86]. LeRoux et al. conducted an exhaustive search of the bacterial genome, ultimately identifying homologs of DarTG, a novel family within TA systems. DarTG1 and DarTG2 were shown to provide strong protection against different phages in *E. coli* MG1655. LeRoux et al. further demonstrated that the release of the DarT toxin, a DNA ADP-ribosyltransferase, is triggered when bacterial cells are infected with RB69 or T5 phages. The toxin subsequently modifies the viral DNA and blocks its replication, thereby effectively preventing the production of mature viral particles [87]. Hsueh et al. revealed a TA (toxin/anti-toxin) system closely related to CBASS, named AvcID, in which AvcD acts as a deoxycytidine deaminase, the enzymatic activity of which is specifically inhibited by a non-coding RNA, AvcI, after the translation process is complete. This unique AvcID system constructs a protective barrier by effectively depleting free deoxycytidine nucleotides when bacteria are infected with phages. This significantly reduces phage replicative activities and provides an important safeguard for bacterial population survival and reproduction [88]. Guo et al. identified a unique kinase–kinase–phosphatase (KKP) system encoded by a prophage in *P. aeruginosa*. Specifically, the KKP manipulator consisted of three core genes, *pfkA*, *pfkB,* and *pfkC*, the expression of which, in *P. aeruginosa* PAO1, did not trigger toxic effects. However, they significantly inhibited cell growth rate and viability when the *pfkA* and *pfkB* genes were co-expressed and then returned to a non-toxic state [89]. The ribonucleic acid exonuclease RNase R, encoded by *rnr* in *Pseudomonas syringae* Lz4W, is important for coping with a wide range of stress conditions, and its inactivation leads to cold sensitization of *P. syringae* Lz4W. The mutant *P. syringae* strain with inactivated *rnr* has an increased copy number of its internal protoplasmid pLz4W at low temperatures, and it has a type II TA system (psA-psT) but poor anti-toxin stability. Degradation of anti-toxins triggers the liberation of toxins, ultimately leading to growth inhibition or cell death. However, the overexpression of the PsA anti-toxin in Δ*rnr* mutants restores cell viability [90].

### 2.7. Bacteriophage Assembly Interference

**Figure 6:**
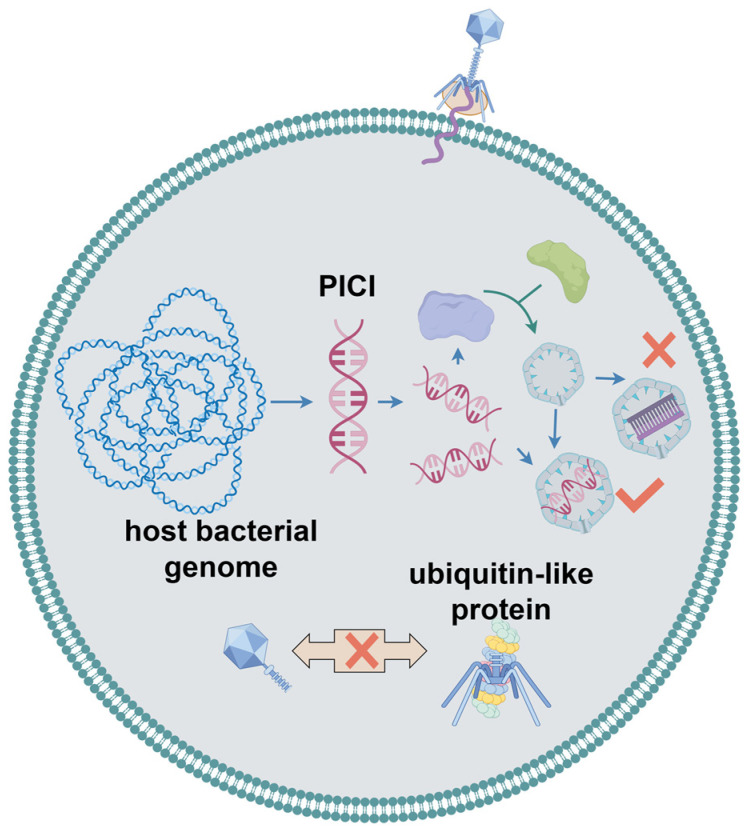
Bacteriophage assembly interference system in bacteria.

Phage-induced chromosomal islands (PICIs) are mobile genetic elements widely found in bacteria [91]. When a Gram-positive bacterium is infected by a helper phage, PICI is cleaved from the bacterial genome, which, in turn, alters the size of the phage capsid and allows preferential packing of the PICI gene cluster. Thus, normal phage assembly was hindered (Figure 6). Unlike in Gram-positive bacteria, PICIs in Gram-negative bacteria are induced by an activator encoded by PICI itself; however, the expression of this activator requires the involvement of helper phages [92,93]. Salom et al. proposed that a PICI is an essential component of the initial bacterial innate immune system [94]. Research on the ubiquitin-like phage defense system proposed by Sorek et al. also showed that the process by which the system specifically binds ubiquitin-like proteins to the central tail fibers of the phage during phage infection is crucial for phage tail assembly. Cells encoding this defense system release partially assembled tail-less phage particles with fewer infections [23].

### 2.8. DRT2 System

**Figure 7:**
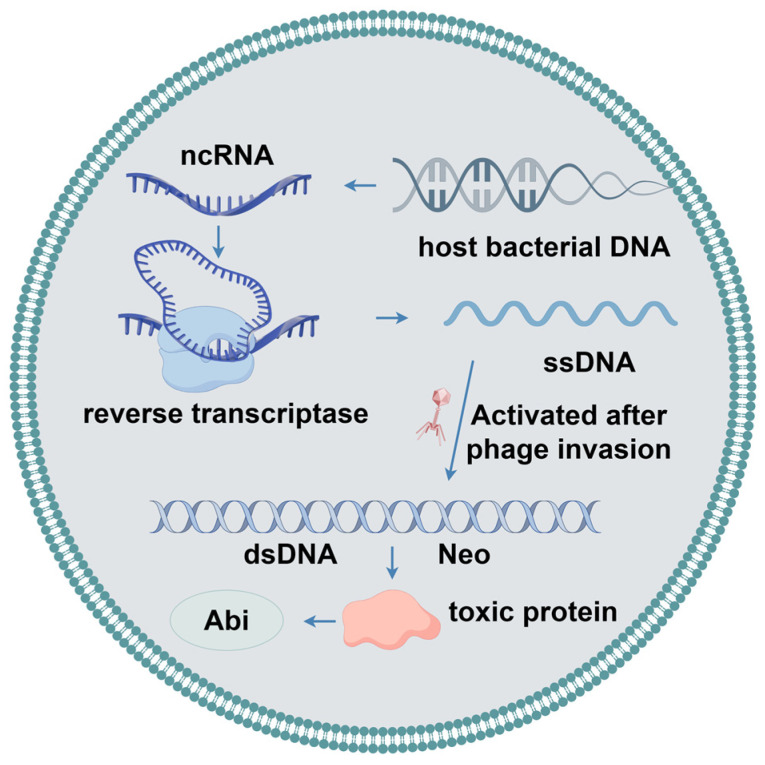
The mechanism of DRT2 system in bacteria.

Recently, a specific defense system within *Klebsiella pneumoniae* named defense-associated reverse transcriptase 2 (DRT2), which contains a reverse transcriptase (RT), was investigated (Figure 7) [95,96]. It was shown that the DRT2 system constituted an extremely effective defense barrier against phages. Unlike conventional phage resistance systems, DRT2 relies on key proteins that are not generated by conventional gene transcription and translation pathways. Although the host bacterium remains uninfected, its internal DNA undergoes a delicate transcription process to produce non-coding RNA (ncRNA). This ncRNA molecule serves as a key mediator for reverse transcription back into single-stranded DNA through an extremely specialized reverse transcription reaction. Different from traditional one-dimensional linear reverse transcription, the DRT2 system employs roll-over reverse transcription to obtain single-stranded DNA. The RT is able to embed the information of the ncRNA template into DNA continuously, generating an extra-long DNA strand including numerous repetitive sequences tightly strung together. When phage successfully invades the host cell, this unique DNA structure activates the synthesis of a second complementary single-stranded DNA, which eventually forms a stable double-stranded DNA product. This double-stranded DNA is not only structurally robust but also possesses a highly efficient transcriptional capacity for producing mRNA encoding a never-ending ORF-Neo without a stop codon. In addition, improved toxicity of Neo will be exhibited as the number of repeats increases. Finally, the transcription of the repetitive mRNA and translation of the repetitive Neo protein will inhibit phage replication, proliferation, and dissemination in the host cell [95,97].

## 3. Anti-Phage Behavior at the Multicellular Level

**Figure 8:**
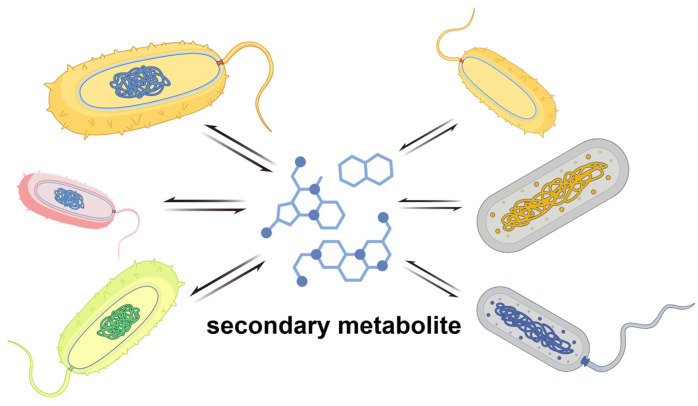
Anti-phage behavior at the multicellular level in bacteria.

Most anti-phage defense systems rely on RNA or protein complexes within individual cells. However, bacteria also exhibit phage resistance at the multicellular level (Figure 8). This multicellular behavior offers a number of advantages, such as improving nutrient acquisition, increasing resistance to physical stress or antimicrobial molecules, and providing protection against parasites [5].

Population sensing is a typical multicellular behavior that can influence the susceptibility of a bacterium to phage infection. For example, it can enhance the effect of the CRISPR-Cas system for target exogenous DNA in *P. aeruginosa* and *Serratia marcescens*, thereby promoting adaptive immunity of CRISPR-Cas at higher cell densities [98]. Environmental bacteria are capable of producing a wide range of biologically active small molecules, many of which play an important role in defense against phages. Kronheim et al. demonstrated that *Streptomyces* usually produces small molecules such as DNA-embedded compounds that can inactivate all invading dsDNA phages [99]. Hardy et al. have summarized the major classes of anti-phage small molecules known to date after in-depth studies, which include anthracyclines, aminoglycosides, and modified nucleotides produced from prokaryotic viperidin. In particular, aminoglycosides exhibited not only significant anti-phage functions, but also potent anti-bacterial effects, which makes them more widely applicable as multifunctional molecules in a variety of fields [100]. It has been shown that Zoerythromycin exerts its specific function during the early stages of the phage replication cycle after DNA translocation but before replication. In addition, adriamycin is able to form free radicals which could directly damage DNA and cause its oxidation, thus producing an effective inhibition of the phage [33].

Otherwise, two compounds, idarubicin and pirubicin, derived from *Streptomyces* also play an important role in the phage life cycle as DNA embedding agents. Specifically, they are able to influence the cyclization process of phage linear DNA or interfere with those key proteins involved in transcription and translation processes, thus effectively inhibiting phage replication and proliferation [99]. Xuan et al. demonstrated that *Shewanella baltica* is unable to produce N-acyl-homoserine lactone (AHLs) signaling molecules; however, it is able to respond to exogenous AHL signaling molecules through its LuxR receptor. Upon receiving this QS signaling molecule, the bacterium regulates phage LPS receptor synthesis by downregulating the expression of *galU* and *tkt* to drive phage resistance [101].

## 4. Application of Phage-Resistant Bacteria Strains

As discussed above, bacteria possess diverse anti-phage mechanisms that provide solid and in-depth theoretical support for constructing efficient anti-phage bacterial strains. For example, *E. coli,* with its clear genetic background, rapid growth, and easy molecular manipulation, has become one of the most popular chassis hosts for the production of natural and non-natural valuable bioproducts [102,103,104]. However, phage contamination has become a major threat to the industrial fermentation of various *E. coli* strains. Therefore, the development of genetically stable strains with strong phage resistance is required. Given that *Lactococcus* phages specifically recognize and bind to host cell surface polysaccharide phosphate (PSP) side chains, Giesbers et al. designed *Lactococcus* mutants by reducing its PSP synthesis. Introducing mutations in the PSP biosynthesis gene cluster drastically reduces PSP production, which in turn triggers phenotypic variation and temporarily weakens the recognition and infection of the phage [105]. However, as a key component of the cell wall, a decrease in intracellular PSP levels results in drastic changes in morphology and interferes with cell growth. To address these issues, Guérin et al. successfully screened spontaneous mutants with restored growth performance and better cell morphology based on *Lactococcus cremoris* PSP-negative mutants. Furthermore, whole-genome sequencing revealed that these mutants carried variants of penicillin-binding protein PBP2b, a key enzyme in peptidoglycan biosynthesis [106]. By reducing or completely inactivating the activity of PBP2b, the growth obstacles and cellular morphology of bacterial cells were significantly improved, while phage resistance was maintained. Wen et al. successfully screened spontaneous phage-insensitive mutants from a population of sensitive strains of *Lactobacillus fermentum*. Compared with the original sensitive strains, these mutant strains not only demonstrated significant resistance to phages, but also showed excellent tolerance to environmental stressors, such as acid and bile salts [107]. Genome resequencing revealed that the mechanisms of phage resistance mainly included interference in the adsorption process and blockage of the DNA injection phase. In addition, a multivalent phage-resistant strain of *E. coli* BL21(DE3) was engineered using a mixture screening strategy. This strain could effectively resist 23 of 32 phages tested while maintaining an enhanced recombinant protein expression level comparable to that of wild-type *E. coli* BL21(DE3) [108].

**Figure 9:**
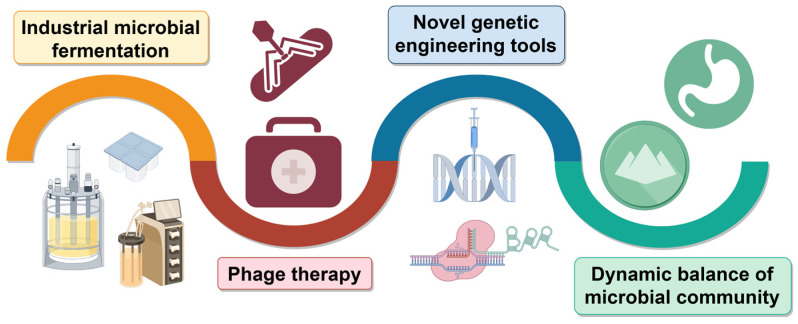
Wide application of phage-resistant bacteria strains.

The potential applications of these strains with unique phage-resistant properties could inspire the development of novel biotherapeutic strategies and antimicrobial agents, opening new avenues for the promotion of human health (Figure 9). *Lactobacilli*, especially *Lactobacillus plantarum*, exhibited extraordinary potential to produce antimicrobial substances that are effective in inhibiting the growth of zoonotic bacterial pathogens. A unique mutant *LP*^+PR^ strain was isolated that exhibited more rapid growth and longer survival time than the phage-sensitive *LP* strain. These excellent characteristics make the mutant *LP*^+PR^ strain a promising candidate for developing feed supplements for farm animals and chassis cells in the fermentation industry [109].

Several pathogenic bacteria exhibit strong resistance to antibiotics. Therefore, phages have become novel tools for the treatment of drug-resistant bacterial infections. Accordingly, an in-depth characterization of the phage resistance mechanism is crucial for guiding the development of phage therapeutic strategies. Xu et al. isolated a *Pseudomonas plecoglossicida* phage vB_PpS_SYP, which showed limited antibacterial effects and was thus difficult to apply directly in phage therapy. To overcome this limitation, they intensively investigated the genetic mechanism of phage-resistant bacteria and identified two crucial mutated genes: GT-1 (glycosyltransferase family 1) and hypothetical outer membrane protein (HomP). By knocking out these two genes, their team found that antibiotics suppress the GT-1 mutant, whereas the evolved phage showed stronger inhibition of the HomP mutant [110]. Further exploration of phage-antibiotic combinations confirmed that combination therapies of chloramphenicol or ciprofloxacin with phage vB_PpS_SYP exhibited superior antimicrobial efficacy compared to monotherapies, opening up a new avenue in drug-resistant strain therapeutics. Similarly, extraordinarily drug-resistant *K. pneumoniae* (ERKp) has been investigated as a possible phage therapy. Although ERKp strains show complete resistance to the antibiotic combination of sulfamethoxazole–methoprene, Bao et al. combined sulfamethoxazole–methoprene with phages to form an innovative combination therapy. This combination effectively inhibits the production of phage-resistant mutants in vitro and successfully cures patients in clinical settings [111]. Zeng et al. successfully isolated a specific strain of *Salmonella enteritidi* designated as sm140, along with its corresponding phage, Psm140, from samples of chicken liver and environmental wastewater. Through an in-depth study, they revealed that mutation of *wbaP* gene in *S. enteritidi* is the key factor in phage resistance [112]. Ellinor et al. performed a full factorial evolutionary experiment aimed at phage evolution in an artificial four-species bacterial community constructed in a laboratory. This result showed that the introduction of phages significantly reshaped the architecture of the microbial community, highlighting the power of phages in regulating microbial community structure [113].

## 5. Conclusions and Outlook

The fermentation industry has attracted increasing attention for its potential to accelerate the transformation from petroleum-based to biomass-centered industrial processes. Owing to the advantages of rapid growth, easy cultivation methods, and sufficient genetic tools, microbial fermentation has been widely applied for the production of dairy products, pharmaceuticals, and other valuable chemicals. However, determining the susceptibility of industrial bacteria to phage contamination is challenging. In nature, diverse phage resistance systems have evolved during the long-term coevolution between phages and their bacterial hosts. By introducing and optimizing these mechanisms in phage-sensitive chassis hosts, recombinant phage-resistant strains, which are desirable for the fermentation industry, can be generated.

However, limitations remain in the construction of phage-resistant strains. The first is the genetic stability of the engineered phage-resistant strains. Phage-resistant strains may suffer from the loss or mutation of resistance elements during genetic transmission, thus weakening or even losing their resistance. Owing to the competition between carbon sources and bacterial growth, product biosynthesis, and biofilm-forming ability, phage-resistant mutant strains often exhibit weakened growth [114]. Second is the potential risk of phage-resistant bacteria. Complex synergistic evolutionary dynamics exist between bacteria and phages. Bacteria can evolve phage-resistant mutations, and phages can be stimulated to evade these new defense mechanisms. Synergistic mutations in phage tail proteins have been shown to be a key strategy for phages to regain their infectivity, and these mutations have endowed phages with the ability to recognize and bind to emerging binding receptors on bacterial mutants or enhance their binding efficiency to potential binding sites, such as the bacterial flagellum [115]. Accordingly, engineering phage-resistant bacteria may disturb the balance of microbial communities in nature, leading to weakened ecosystem stability. Third is the limitations in the resistance mechanisms. The bactericidal efficacy of phages is highly specific to a particular host strain, whereas bacterial resistance to phages is not absolute and is often lost owing to subtle mutations in the phage [116]. Phage-resistant strains tend to be targeted and effective against specific phages [117]. They exhibit rapid replication, large burst sizes, and genomic adaptability. On the other hand, phages can rapidly generate resistance to existing defense systems through mutations and recombination. For example, the invasion of phage lambda was dependent on the LamB receptor on the surface of *E. coli*. When the expression of the LamB receptor was suppressed by genetic mutation, phage lambda gradually evolved the ability to recognize another receptor, OmpF [33]. Annoj et al. also showed that lysogenic phages in *P. aeruginosa* could express anti-CRISPR proteins interfering with the CRISPR-Cas system. As a result, the assembly of the Cas9 complex was inhibited [118]. In addition, the Dmd protein was generated when phage T4 began its infection process in *E. coli*. This protein could inhibit the toxin activity directly and protect the host cell from imminent death [119]. This intrinsic limitation of the resistance mechanism significantly weakens the defense of strains against diverse phages, making them particularly vulnerable [120].

Several strategies have been proposed to overcome these challenges. First is the exploration and validation of novel phage-resistance mechanisms using public databases. These novel systems provide a potential solution for phage contamination during industrial fermentation processes, especially for phages insensitive to present anti-phage mechanisms. However, an increasing number of anti-phage mechanisms will facilitate the elucidation of interactions between bacterial hosts and phages. Second, a single defense system is often insufficient to defend against complex populations of contaminating phages. Once the defense system against phage infection is delayed or weakened, phages can easily evolve escape mechanisms through rapid mutation and massive reproduction. Accordingly, a combination of more than one phage resistance mechanism in a chassis host may be effective. To alleviate the competition between strain growth and the expression of anti-phage elements, the utilization of synthetic biology circuits, such as growth-coupled and multilayered dynamic regulation networks [121] and growth biosensors [122] can be beneficial. As the overexpression of anti-phage mechanisms often generates an additional metabolic burden for the host strain, each microorganism within a population can be responsible for a unique defense mechanism. Consequently, multilayered phage-defense networks can be constructed. When facing phage infection, the constructed bacterial communities can fine-tune their defense libraries via mobile genetic elements and horizontal gene transfer [65].

The continuous emergence of drug-resistant bacteria poses a serious threat to public health, whereas phages, as potential antimicrobial tools, are limited by host specificity. With the rapid development of gene editing technology, scientists are attempting to improve the bactericidal effect of phages through genetic modifications and to reduce the risk of triggering bacterial resistance. In addition, the proposal of phage cocktail therapy, which expands the antimicrobial spectrum by combining multiple phages, provides new ideas for dealing with complex infections and drug-resistant strains. The combination of phages and antibiotics is also regarded as a promising therapeutic strategy aimed at slowing the development of bacterial resistance. In addition, combined therapy (phages plus drugs) has a notable effect in eliminating both phage- and drug-resistant bacteria [123,124]. With the rapid progress in synthetic biology and global surveillance networks for phage monitoring, the development of phage-resistant bacteria can be further accelerated, facilitating a new era of biomanufacturing.

## Data Availability

Not applicable.

## References

[1] Suttle C.A. (2007). Marine viruses—Major players in the global ecosystem. Nat. Rev. Microbiol..

[2] Simmonds P., Adriaenssens E.M., Zerbini F.M., Abrescia N.G.A., Aiewsakun P., Alfenas-Zerbini P., Bao Y., Barylski J., Drosten C., Duffy S. (2023). Four principles to establish a universal virus taxonomy. PLoS Biol..

[3] Martínez-Borra J., González S., López-Larrea C. (2012). The origin of the bacterial immune response. Adv. Exp. Med. Biol..

[4] Vassallo C.N., Doering C.R., Littlehale M.L., Teodoro G.I.C., Laub M.T. (2022). A functional selection reveals previously undetected anti-phage defence systems in the *E. coli* pangenome. Nat. Microbiol..

[5] Luthe T., Kever L., Thormann K., Frunzke J. (2023). Bacterial multicellular behavior in antiviral defense. Curr. Opin. Microbiol..

[6] Rostøl J.T., Marraffini L. (2019). (Ph)ighting Phages: How Bacteria Resist Their Parasites. Cell Host Microbe.

[7] Arias C.F., Acosta F.J., Bertocchini F., Herrero M.A., Fernández-Arias C. (2022). The coordination of anti-phage immunity mechanisms in bacterial cells. Nat. Commun..

[8] Zou X., Xiao X.H., Mo Z.R., Ge Y.S., Jiang X., Huang R.L., Li M.X., Deng Z.X., Chen S., Wang L.R. (2022). Systematic strategies for developing phage resistant *Escherichia coli* strains. Nat. Commun..

[9] Karpov D.S. (2024). CRISPR-Cas Systems and Genome Editing: Beginning the Era of CRISPR/Cas Therapies for Humans. Int. J. Mol. Sci..

[10] Chen Y., Batra H., Dong J., Chen C., Rao V.B., Tao P. (2019). Genetic Engineering of Bacteriophages Against Infectious Diseases. Front. Microbiol..

[11] Domingo-Calap P., Mora-Quilis L., Sanjuán R. (2020). Social Bacteriophages. Microorganisms.

[12] Reyes-Robles T., Dillard R.S., Cairns L.S., Silva-Valenzuela C.A., Housman M., Ali A., Wright E.R., Camilli A., DiRita V.J. (2018). *Vibrio cholerae* Outer Membrane Vesicles Inhibit Bacteriophage Infection. J. Bacteriol..

[13] Zhang J., Li W., Zhang Q., Wang H., Xu X., Diao B., Zhang L., Kan B. (2009). The core oligosaccharide and thioredoxin of *Vibrio cholerae* are necessary for binding and propagation of its typing phage VP3. J. Bacteriol..

[14] Pickard D., Toribio A.L., Petty N.K., van Tonder A., Yu L., Goulding D., Barrell B., Rance R., Harris D., Wetter M. (2010). A conserved acetyl esterase domain targets diverse bacteriophages to the Vi capsular receptor of *Salmonella enterica* Serovar Typhi. J. Bacteriol..

[15] Harvey H., Bondy-Denomy J., Marquis H., Sztanko K.M., Davidson A.R., Burrows L.L. (2018). *Pseudomonas aeruginosa* defends against phages through type IV pilus glycosylation. Nat. Microbiol..

[16] Trudelle D.M., Bryan D.W., Hudson L.K., Denes T.G. (2019). Cross-resistance to phage infection in *Listeria monocytogenes* serotype 1/2a mutants. Food Microbiol..

[17] Scholl D., Adhya S., Merril C. (2005). *Escherichia coli* K1’s capsule is a barrier to bacteriophage T7. Appl. Environ. Microbiol..

[18] Burmeister A.R., Fortier A., Roush C., Lessing A.J., Bender R.G., Barahman R., Grant R., Chan B.K., Turner P.E. (2020). Pleiotropy complicates a trade-off between phage resistance and antibiotic resistance. Proc. Natl. Acad. Sci. USA.

[19] Xuan G., Lin H., Kong J., Wang J. (2022). Phage Resistance Evolution Induces the Sensitivity of Specific Antibiotics in *Pseudomonas aeruginosa* PAO1. Microbiol. Spectr..

[20] Endriss F., Braun V. (2004). Loop deletions indicate regions important for FhuA transport and receptor functions in *Escherichia coli*. J. Bacteriol..

[21] Riede I., Eschbach M.L. (1986). Evidence that TraT interacts with OmpA of *Escherichia coli*. FEBS Lett..

[22] Achtman M., Kennedy N., Skurray R. (1977). Cell—Cell interactions in conjugating *Escherichia coli*: Role of *traT* protein in surface exclusion. Proc. Natl. Acad. Sci. USA.

[23] Hor J., Wolf S.G., Sorek R. (2024). Bacteria conjugate ubiquitin-like proteins to interfere with phage assembly. Nature.

[24] van Houte S., Buckling A., Westra E.R. (2016). Evolutionary Ecology of Prokaryotic Immune Mechanisms. Microbiol. Mol. Biol. Rev..

[25] Labrie S.J., Samson J.E., Moineau S. (2010). Bacteriophage resistance mechanisms. Nat. Rev. Microbiol..

[26] Wang W.Q., Li Y.M., Tang K.H., Lin J.Z., Gao X.Y., Guo Y.X., Wang X.X. (2022). Filamentous prophage capsid proteins contribute to superinfection exclusion and phage defence in *Pseudomonas aeruginosa*. Environ. Microbiol..

[27] Shi K., Oakland J.T., Kurniawan F., Moeller N.H., Banerjee S., Aihara H. (2020). Structural basis of superinfection exclusion by bacteriophage T4 Spackle. Commun. Biol..

[28] Sun X., Göhler A., Heller K.J., Neve H. (2006). The *ltp* gene of temperate *Streptococcus thermophilus* phage TP-J34 confers superinfection exclusion to *Streptococcus thermophilus* and *Lactococcus lactis*. Virology.

[29] Illingworth C., Hunter M., Fusco D. (2022). Superinfection exclusion: A viral strategy with short-term benefits and long-term drawbacks. PLoS Comput. Biol..

[30] Georjon H., Bernheim A. (2023). The highly diverse antiphage defence systems of bacteria. Nat. Rev. Microbiol..

[31] Tesson F., Hervé A., Mordret E., Touchon M., d’Humières C., Cury J., Bernheim A. (2022). Systematic and quantitative view of the antiviral arsenal of prokaryotes. Nat. Commun..

[32] Tock M.R., Dryden D.T.F. (2005). The biology of restriction and anti-restriction. Curr. Opin. Microbiol..

[33] Teklemariam A.D., Al-Hindi R.R., Qadri I., Alharbi M.G., Ramadan W.S., Ayubu J., Al-Hejin A.M., Hakim R.F., Hakim F.F., Hakim R.F. (2023). The Battle between Bacteria and Bacteriophages: A Conundrum to Their Immune System. Antibiotics.

[34] Dy R.L., Richter C., Salmond G.P.C., Fineran P.C. (2014). Remarkable Mechanisms in Microbes to Resist Phage Infections. Annu. Rev. Virol..

[35] Goldfarb T., Sberro H., Weinstock E., Cohen O., Doron S., Charpak-Amikam Y., Afik S., Ofir G., Sorek R. (2014). BREX is a novel phage resistance system widespread in microbial genomes. EMBO J..

[36] Gordeeva J., Morozova N., Sierro N., Isaev A., Sinkunas T., Tsvetkova K., Matlashov M., Truncaitė L., Morgan R.D., Ivanov N.V. (2019). BREX system of *Escherichia coli* distinguishes self from non-self by methylation of a specific DNA site. Nucleic Acids Res..

[37] Ofir G., Melamed S., Sberro H., Mukamel Z., Silverman S., Yaakov G., Doron S., Sorek R. (2017). DISARM is a widespread bacterial defence system with broad anti-phage activities. Nat. Microbiol..

[38] Weigele P., Raleigh E.A. (2016). Biosynthesis and Function of Modified Bases in Bacteria and Their Viruses. Chem. Rev..

[39] Sun Y., Kong L., Wu G., Cao B., You D. (2020). DNA Phosphorothioate Modifications Are Widely Distributed in the Human Microbiome. Biomolecules.

[40] Xiong L., Liu S., Chen S., Xiao Y., Zhu B., Gao Y., Zhang Y., Chen B., Luo J., Deng Z. (2019). A new type of DNA phosphorothioation-based antiviral system in archaea. Nat. Commun..

[41] Xiong X., Wu G., Wei Y., Liu L., Zhang Y., Su R., Jiang X., Li M., Gao H., Tian X. (2020). SspABCD-SspE is a phosphorothioation-sensing bacterial defence system with broad anti-phage activities. Nat. Microbiol..

[42] Wang S., Wan M., Huang R., Zhang Y., Xie Y., Wei Y., Ahmad M., Wu D., Hong Y., Deng Z. (2021). SspABCD-SspFGH Constitutes a New Type of DNA Phosphorothioate-Based Bacterial Defense System. mBio.

[43] Tong T., Chen S., Wang L., Tang Y., Ryu J.Y., Jiang S., Wu X., Chen C., Luo J., Deng Z. (2018). Occurrence, evolution, and functions of DNA phosphorothioate epigenetics in bacteria. Proc. Natl. Acad. Sci. USA.

[44] Hille F., Richter H., Wong S.P., Bratovič M., Ressel S., Charpentier E. (2018). The Biology of CRISPR-Cas: Backward and Forward. Cell.

[45] Makarova K.S., Wolf Y.I., Iranzo J., Shmakov S.A., Alkhnbashi O.S., Brouns S.J.J., Charpentier E., Cheng D., Haft D.H., Horvath P. (2020). Evolutionary classification of CRISPR-Cas systems: A burst of class 2 and derived variants. Nat. Rev. Microbiol..

[46] Koonin E.V., Makarova K.S., Zhang F. (2017). Diversity, classification and evolution of CRISPR-Cas systems. Curr. Opin. Microbiol..

[47] Nami Y., Rostampour M., Panahi B. (2023). CRISPR-Cas systems and diversity of targeting phages in *Lactobacillus johnsonii* strains; insights from genome mining approach. Infect. Genet. Evol..

[48] Deng X., Yuan J., Chen L., Chen H., Wei C., Nielsen P.H., Wuertz S., Qiu G. (2023). CRISPR-Cas phage defense systems and prophages in *Candidatus* Accumulibacter. Water Res..

[49] Lin P., Pu Q., Shen G., Li R., Guo K., Zhou C., Liang H., Jiang J., Wu M. (2019). CdpR Inhibits CRISPR-Cas Adaptive Immunity to Lower Anti-viral Defense while Avoiding Self-Reactivity. iScience.

[50] Cury J., Bernheim A. (2022). CRISPR-Cas and restriction–modification team up to achieve long-term immunity. Trends Microbiol..

[51] Lopatina A., Tal N., Sorek R. (2020). Abortive Infection: Bacterial Suicide as an Antiviral Immune Strategy. Annu. Rev. Virol..

[52] Depardieu F., Didier J.P., Bernheim A., Sherlock A., Molina H., Duclos B., Bikard D. (2016). A Eukaryotic-like Serine/Threonine Kinase Protects Staphylococci against Phages. Cell Host Microbe.

[53] Garb J., Lopatina A., Bernheim A., Zaremba M., Siksnys V., Melamed S., Leavitt A., Millman A., Amitai G., Sorek R. (2022). Multiple phage resistance systems inhibit infection via SIR2-dependent NAD(+) depletion. Nat. Microbiol..

[54] Zaremba M., Dakineviciene D., Golovinas E., Zagorskaitė E., Stankunas E., Lopatina A., Sorek R., Manakova E., Ruksenaite A., Silanskas A. (2022). Short prokaryotic Argonautes provide defence against incoming mobile genetic elements through NAD(+) depletion. Nat. Microbiol..

[55] Sather L.M., Zamani M., Muhammed Z., Kearsley J.V.S., Fisher G.T., Jones K.M., Finan T.M. (2023). A broadly distributed predicted helicase/nuclease confers phage resistance via abortive infection. Cell Host Microbe.

[56] Mayo-Muñoz D., Smith L.M., Garcia-Doval C., Malone L.M., Harding K.R., Jackson S.A., Hampton H.G., Fagerlund R.D., Gumy L.F., Fineran P.C. (2022). Type III CRISPR-Cas provides resistance against nucleus-forming jumbo phages via abortive infection. Mol. Cell.

[57] Millman A., Bernheim A., Stokar-Avihail A., Fedorenko T., Voichek M., Leavitt A., Oppenheimer-Shaanan Y., Sorek R. (2020). Bacterial Retrons Function In Anti-Phage Defense. Cell.

[58] Bobonis J., Mitosch K., Mateus A., Karcher N., Kritikos G., Selkrig J., Zietek M., Monzon V., Pfalz B., Garcia-Santamarina S. (2022). Bacterial retrons encode phage-defending tripartite toxin-antitoxin systems. Nature.

[59] Tal N., Millman A., Stokar-Avihail A., Fedorenko T., Leavitt A., Melamed S., Yirmiya E., Avraham C., Brandis A., Mehlman T. (2022). Bacteria deplete deoxynucleotides to defend against bacteriophage infection. Nat. Microbiol..

[60] Margolis S.R., Wilson S.C., Vance R.E. (2017). Evolutionary Origins of cGAS-STING Signaling. Trends Immunol..

[61] Cohen D., Melamed S., Millman A., Shulman G., Oppenheimer-Shaanan Y., Kacen A., Doron S., Amitai G., Sorek R. (2019). Cyclic GMP-AMP signalling protects bacteria against viral infection. Nature.

[62] Davies B.W., Bogard R.W., Young T.S., Mekalanos J.J. (2012). Coordinated regulation of accessory genetic elements produces cyclic di-nucleotides for *V. cholerae* virulence. Cell.

[63] Lowey B., Whiteley A.T., Keszei A.F.A., Morehouse B.R., Mathews I.T., Antine S.P., Cabrera V.J., Kashin D., Niemann P., Jain M. (2020). CBASS Immunity Uses CARF-Related Effectors to Sense 3′-5′- and 2′-5′-Linked Cyclic Oligonucleotide Signals and Protect Bacteria from Phage Infection. Cell.

[64] Whiteley A.T., Eaglesham J.B., de Oliveira Mann C.C., Morehouse B.R., Lowey B., Nieminen E.A., Danilchanka O., King D.S., Lee A.S.Y., Mekalanos J.J. (2019). Bacterial cGAS-like enzymes synthesize diverse nucleotide signals. Nature.

[65] Bernheim A., Sorek R. (2020). The pan-immune system of bacteria: Antiviral defence as a community resource. Nat. Rev. Microbiol..

[66] Millman A., Melamed S., Amitai G., Sorek R. (2020). Diversity and classification of cyclic-oligonucleotide-based anti-phage signalling systems. Nat. Microbiol..

[67] Ye Q., Lau R.K., Mathews I.T., Birkholz E.A., Watrous J.D., Azimi C.S., Pogliano J., Jain M., Corbett K.D. (2020). HORMA Domain Proteins and a Trip13-like ATPase Regulate Bacterial cGAS-like Enzymes to Mediate Bacteriophage Immunity. Mol. Cell.

[68] Duncan-Lowey B., McNamara-Bordewick N.K., Tal N., Sorek R., Kranzusch P.J. (2021). Effector-mediated membrane disruption controls cell death in CBASS antiphage defense. Mol. Cell.

[69] Lau R.K., Ye Q., Birkholz E.A., Berg K.R., Patel L., Mathews I.T., Watrous J.D., Ego K., Whiteley A.T., Lowey B. (2020). Structure and Mechanism of a Cyclic Trinucleotide-Activated Bacterial Endonuclease Mediating Bacteriophage Immunity. Mol. Cell.

[70] Gao L., Altae-Tran H., Böhning F., Makarova K.S., Segel M., Schmid-Burgk J.L., Koob J., Wolf Y.I., Koonin E.V., Zhang F. (2020). Diverse enzymatic activities mediate antiviral immunity in prokaryotes. Science.

[71] Burroughs A.M., Zhang D., Schäffer D.E., Iyer L.M., Aravind L. (2015). Comparative genomic analyses reveal a vast, novel network of nucleotide-centric systems in biological conflicts, immunity and signaling. Nucleic Acids Res..

[72] Makarova K.S., Gao L., Zhang F., Koonin E.V. (2019). Unexpected connections between type VI-B CRISPR-Cas systems, bacterial natural competence, ubiquitin signaling network and DNA modification through a distinct family of membrane proteins. FEMS Microbiol. Lett..

[73] Gao Y., Luo X., Li P., Li Z., Ye F., Liu S., Gao P. (2023). Molecular basis of RADAR anti-phage supramolecular assemblies. Cell.

[74] Duncan-Lowey B., Tal N., Johnson A.G., Rawson S., Mayer M.L., Doron S., Millman A., Melamed S., Fedorenko T., Kacen A. (2023). Cryo-EM structure of the RADAR supramolecular anti-phage defense complex. Cell.

[75] Page R., Peti W. (2016). Toxin-antitoxin systems in bacterial growth arrest and persistence. Nat. Chem. Biol..

[76] Lin J., Guo Y., Yao J., Tang K., Wang X. (2023). Applications of toxin-antitoxin systems in synthetic biology. Eng. Microbiol..

[77] Song S., Wood T.K. (2020). A Primary Physiological Role of Toxin/Antitoxin Systems Is Phage Inhibition. Front. Microbiol..

[78] Qiu J., Zhai Y., Wei M., Zheng C., Jiao X. (2022). Toxin-antitoxin systems: Classification, biological roles, and applications. Microbiol. Res..

[79] Guegler C.K., Laub M.T. (2021). Shutoff of host transcription triggers a toxin-antitoxin system to cleave phage RNA and abort infection. Mol. Cell.

[80] Dy R.L., Przybilski R., Semeijn K., Salmond G.P., Fineran P.C. (2014). A widespread bacteriophage abortive infection system functions through a Type IV toxin-antitoxin mechanism. Nucleic Acids Res..

[81] Cui Y., Su X., Wang C., Xu H., Hu D., Wang J., Pei K., Sun M., Zou T. (2022). Bacterial MazF/MazE toxin-antitoxin suppresses lytic propagation of arbitrium-containing phages. Cell Rep..

[82] Songailiene I., Juozapaitis J., Tamulaitiene G., Ruksenaite A., Šulčius S., Sasnauskas G., Venclovas Č., Siksnys V. (2020). HEPN-MNT Toxin-Antitoxin System: The HEPN Ribonuclease Is Neutralized by OligoAMPylation. Mol. Cell.

[83] Hoskisson P.A., Sumby P., Smith M.C.M. (2015). The phage growth limitation system in *Streptomyces coelicolor* A(3)2 is a toxin/antitoxin system, comprising enzymes with DNA methyltransferase, protein kinase and ATPase activity. Virology.

[84] Czarnecki J., Dziewit L., Kowalski L., Ochnio M., Bartosik D. (2015). Maintenance and genetic load of plasmid pKON1 of *Paracoccus kondratievae*, containing a highly efficient toxin-antitoxin module of the *hipAB* family. Plasmid.

[85] Chen Z., Yao J., Zhang P., Wang P., Ni S., Liu T., Zhao Y., Tang K., Sun Y., Qian Q. (2023). Minimized antibiotic-free plasmid vector for gene therapy utilizing a new toxin-antitoxin system. Metab. Eng..

[86] Bleriot I., Blasco L., Pacios O., Fernández-García L., Ambroa A., López M., Ortiz-Cartagena C., Cuenca F.F., Oteo-Iglesias J., Pascual Á. (2022). The role of PemIK (PemK/PemI) type II TA system from *Klebsiella pneumoniae* clinical strains in lytic phage infection. Sci. Rep..

[87] LeRoux M., Srikant S., Teodoro G.I.C., Zhang T., Littlehale M.L., Doron S., Badiee M., Leung A.K.L., Sorek R., Laub M.T. (2022). The DarTG toxin-antitoxin system provides phage defence by ADP-ribosylating viral DNA. Nat. Microbiol..

[88] Hsueh B.Y., Severin G.B., Elg C.A., Waldron E.J., Kant A., Wessel A.J., Dover J.A., Rhoades C.R., Ridenhour B.J., Parent K.N. (2022). Phage defence by deaminase-mediated depletion of deoxynucleotides in bacteria. Nat. Microbiol..

[89] Guo Y., Tang K., Sit B., Gu J., Chen R., Lin J., Lin S., Liu X., Wang W., Gao X. (2022). Dual control of lysogeny and phage defense by a phosphorylation-based toxin/antitoxin system. bioRxiv.

[90] Mittal P., Sinha A.K., Pandiyan A., Kumari L., Ray M.K., Pavankumar T.L. (2024). A type II toxin-antitoxin system is responsible for the cell death at low temperature in *Pseudomonas syringae* Lz4W lacking RNase R. J. Biol. Chem..

[91] Fillol-Salom A., Martínez-Rubio R., Abdulrahman R.F., Chen J., Davies R., Penadés J.R. (2018). Phage-inducible chromosomal islands are ubiquitous within the bacterial universe. ISME J..

[92] Penadés J.R., Christie G.E. (2015). The Phage-Inducible Chromosomal Islands: A Family of Highly Evolved Molecular Parasites. Annu. Rev. Virol..

[93] Tormo-Más M.A., Mir I., Shrestha A., Tallent S.M., Campoy S., Lasa I., Barbé J., Novick R.P., Christie G.E., Penadés J.R. (2010). Moonlighting bacteriophage proteins derepress staphylococcal pathogenicity islands. Nature.

[94] Fillol-Salom A., Miguel-Romero L., Marina A., Chen J., Penadés J.R. (2020). Beyond the CRISPR-Cas safeguard: PICI-encoded innate immune systems protect bacteria from bacteriophage predation. Curr. Opin. Microbiol..

[95] Tang S., Conte V., Zhang D.J., Žedaveinytė R., Lampe G.D., Wiegand T., Tang L.C., Wang M., Walker M.W.G., George J.T. (2024). De novo gene synthesis by an antiviral reverse transcriptase. Science.

[96] Wilkinson M.E., Li D., Gao A., Macrae R.K., Zhang F. (2024). Phage-triggered reverse transcription assembles a toxic repetitive gene from a noncoding RNA. Science.

[97] Osterman I., Sorek R. (2024). Tricking phages with a reverse move. Science.

[98] Høyland-Kroghsbo N.M., Paczkowski J., Mukherjee S., Broniewski J., Westra E., Bondy-Denomy J., Bassler B.L. (2017). Quorum sensing controls the *Pseudomonas aeruginosa* CRISPR-Cas adaptive immune system. Proc. Natl. Acad. Sci. USA.

[99] Kronheim S., Daniel-Ivad M., Duan Z., Hwang S., Wong A.I., Mantel I., Nodwell J.R., Maxwell K.L. (2018). A chemical defence against phage infection. Nature.

[100] Hardy A., Kever L., Frunzke J. (2023). Antiphage small molecules produced by bacteria-beyond protein-mediated defenses. Trends Microbiol..

[101] Xuan G., Tan L., Yang Y., Kong J., Lin H., Wang J. (2023). Quorum sensing autoinducers AHLs protect *Shewanella baltica* against phage infection. Int. J. Food Microbiol..

[102] Mienda B.S., Drager A. (2021). Genome-Scale Metabolic Modeling of *Escherichia coli* and Its Chassis Design for Synthetic Biology Applications. Methods Mol. Biol..

[103] Dong X.R., Liu B., Bao Y.H., Liu W.F., Tao Y. (2023). Metabolic engineering of *Escherichia coli* for high-level production of violaxanthin. Microb. Cell Fact..

[104] Bao Z.X., Gao Y.T., Song Y.T., Ding N., Li W., Wu Q., Zhang X.M., Zheng Y., Li J.M., Hu X.J. (2024). Construction of an *Escherichia coli* chassis for efficient biosynthesis of human-like *N*-linked glycoproteins. Front. Bioeng. Biotechnol..

[105] Giesbers C.A.P., Fagan J., Parlindungan E., Palussiere S., Courtin P., Lugli G.A., Ventura M., Kulakauskas S., Chapot-Chartier M.-P., Mahony J. (2023). Reduced synthesis of phospho-polysaccharide in *Lactococcus* as a strategy to evade phage infection. Int. J. Food Microbiol..

[106] Guérin H., Quénée P., Palussière S., Courtin P., André G., Péchoux C., Costache V., Mahony J., van Sinderen D., Kulakauskas S. (2023). PBP2b Mutations Improve the Growth of Phage-Resistant *Lactococcus cremoris* Lacking Polysaccharide Pellicle. Appl. Environ. Microbiol..

[107] Wen Q., Chen X., Xu M., Liu R., Lian W., Ma Y., Ibrahim A.A. (2024). Selection and characterization of spontaneous phage-resistant mutant of *Limosilactobacillus fermentum*. Int. J. Food Microbiol..

[108] Li P., Lin H., Mi Z., Xing S., Tong Y., Wang J. (2019). Screening of Polyvalent Phage-Resistant *Escherichia coli* Strains Based on Phage Receptor Analysis. Front. Microbiol..

[109] Nagarajan V., Peng M., Tabashsum Z., Salaheen S., Padilla J., Biswas D. (2019). Antimicrobial Effect and Probiotic Potential of Phage Resistant *Lactobacillus plantarum* and its Interactions with Zoonotic Bacterial Pathogens. Foods.

[110] Xu Z., Ding Z., Shi L., Xie Y., Zhang Y., Sao S., Wang Q., Liu Q. (2023). Design combinations of evolved phage and antibiotic for antibacterial guided by analyzing the phage resistance of poorly antimicrobial phage. Microbiol. Spectr..

[111] Bao J., Wu N.N., Zeng Y.G., Chen L.G., Li L.L., Yang L., Zhang Y.Y., Guo M.Q., Li L.S., Li J. (2020). Non-active antibiotic and bacteriophage synergism to successfully treat recurrent urinary tract infection caused by extensively drug-resistant *Klebsiella pneumoniae*. Emerg. Microbes Infect..

[112] Zeng Y.K., Shen M.M., Liu S.L., Zhou X. (2024). Characterization and resistance mechanism of phage-resistant strains of *Salmonella enteritidis*. Poult. Sci..

[113] Alseth E.O., Custodio R., Sundius S.A., Kuske R.A., Brown S.P., Westra E.R. (2024). The impact of phage and phage resistance on microbial community dynamics. PLoS Biol..

[114] Zeng X., Liang S., Dong J., Gao G., Hu Y., Sun Y. (2024). The trade-off of *Vibrio parahaemolyticus* between bacteriophage resistance and growth competitiveness. Front. Microbiol..

[115] Yuan Y., Peng Q., Zhang S., Liu T., Yang S., Yu Q., Wu Y., Gao M. (2019). Phage Reduce Stability for Regaining Infectivity during Antagonistic Coevolution with Host Bacterium. Viruses.

[116] Su-Jin P., Han J.-h., Kim Y.-K. (2016). Isolation of bacteriophage-resistant *Pseudomonas tolaasii* strains and their pathogenic characters. J. Appl. Biol. Chem..

[117] Sorensen P.E., Baig S., Stegger M., Ingmer H., Garmyn A., Butaye P. (2021). Spontaneous Phage Resistance in Avian Pathogenic *Escherichia coli*. Front Microbiol..

[118] Thavalingam A., Cheng Z., Garcia B., Huang X., Shah M., Sung W., Wang M., Harrington L., Hwang S., Hidalgo-Reyes Y. (2019). Inhibition of CRISPR-Cas9 ribonucleoprotein complex assembly by anti-CRISPR AcrIIC2. Nat. Commun..

[119] Otsuka Y., Yonesaki T. (2012). Dmd of bacteriophage T4 functions as an antitoxin against *Escherichia coli* LsoA and RnlA toxins. Mol. Microbiol..

[120] Costa A.R., van den Berg D.F., Esser J.Q., Muralidharan A., van den Bossche H., Bonilla B.E., van der Steen B.A., Haagsma A.C., Fluit A.C., Nobrega F.L. (2024). Accumulation of defense systems in phage-resistant strains of *Pseudomonas aeruginosa*. Sci. Adv..

[121] Zhou S., Yuan S.-F., Nair P.H., Alper H.S., Deng Y., Zhou J. (2021). Development of a growth coupled and multi-layered dynamic regulation network balancing malonyl-CoA node to enhance (2*S*)-naringenin biosynthesis in *Escherichia coli*. Metab. Eng..

[122] Schann K., Bakker J., Boinot M., Kuschel P., He H., Nattermann M., Paczia N., Erb T., Bar-Even A., Wenk S. (2024). Design, construction and optimization of formaldehyde growth biosensors with broad application in biotechnology. Microb. Biotechnol..

[123] Diallo K., Dublanchet A. (2022). Benefits of Combined Phage-Antibiotic Therapy for the Control of Antibiotic-Resistant Bacteria: A Literature Review. Antibiotics.

[124] Necel A., Bloch S., Topka-Bielecka G., Janiszewska A., Łukasiak A., Nejman-Faleńczyk B., Węgrzyn G. (2022). Synergistic Effects of Bacteriophage vB_Eco4-M7 and Selected Antibiotics on the Biofilm Formed by Shiga Toxin-Producing *Escherichia coli*. Antibiotics.

